# Perturbations of Adjuvant Chemotherapy on Cardiovascular Responses and Exercise Tolerance in Patients with Early-Stage Breast Cancer

**DOI:** 10.3390/biology10090910

**Published:** 2021-09-13

**Authors:** Hsin-Fu Lin, Ching-Ying Tseng, Toby Mündel, Yi-Yuan Lin, Chung-Chi Lin, Chiao-Nan Chen, Yi-Hung Liao

**Affiliations:** 1 Department of Athletics, National Taiwan University, Taipei 10617, Taiwan; hsinfu@ntu.edu.tw; 2 Division of Rehabilitation Medicine—Physical Therapy, Koo Foundation Sun Yat-Sen Cancer Center, Taipei 11259, Taiwan; tseng6510@gmail.com; 3 School of Sport, Exercise and Nutrition, Massey University, Palmerston North 4474, New Zealand; T.Mundel@massey.ac.nz; 4 Department of Exercise and Health Science, National Taipei University of Nursing and Health Sciences, Taipei 11219, Taiwan; yiyuanlin.alison@gmail.com (Y.-Y.L.); Cclin20@gmail.com (C.-C.L.); 5 Healthcare and Management Center, Taipei Veterans General Hospital, Taipei 11219, Taiwan; 6 Department of Physical Therapy and Assistive Technology, National Yang Ming Chiao Tung University, Taipei 11221, Taiwan

**Keywords:** doxorubicin, taxanes, CAF, AC-T, cardiovascular responses, SEVR

## Abstract

**Simple Summary:**

The present study aimed to assess and compare the effects of receiving CAF (cyclophosphamide/doxorubicin/fluorouracil) and AC-T (doxorubicin/cyclophosphamide→taxanes) on exercise tolerance and cardiovascular responses in patients with early-stage breast cancer. We herein demonstrated that AC-T chemotherapy increased resting heart rate (RHR) and induced a greater reduction in exercise tolerance at the end of chemotherapy compared with CAF. Moreover, AC-T also lowered myocardial perfusion more than CAF, and it appeared that myocardial impairment occurred before the development of arterial stiffening after chemotherapy. We, therefore, suggest that AC-T chemotherapy might further limit the exercise capacity of patients with early-stage breast cancer. This study provides fundamental information regarding the variety of cardiovascular responses to exercise after chemotherapy in patients with early-stage breast cancer. This information will help clinical professionals in the fields of oncological and rehabilitation medicine to precisely prescribe post-chemotherapy exercise programs when patients are receiving different chemotherapies.

**Abstract:**

Background: Adjuvant chemotherapies are commonly used for treating early-stage breast cancer. However, whether chemotherapeutic regimens affect exercise tolerance and cardiovascular responses remains unclear. Therefore, we investigated the effects of receiving CAF and AC-T on exercise tolerance and cardiovascular responses in patients with early-stage breast cancer. Methods: Thirty-four patients with breast cancer (age: 44 ± 1 years; stage I-II) received either CAF (*n* = 15) or AC-T (*n* = 19), depending on clinical decisions. Their step-exercise tolerance and cardiovascular responses were assessed before and after chemotherapy. Results: After chemotherapy, there were no differences in baseline measurements between patients receiving CAF or AC-T. The increases in resting heart rate (RHR) of those receiving AC-T was significantly greater than that of those receiving CAF. CAF and AC-T did not result in increased pulse wave velocity (PWV), yet the subendocardial viability ratio (SEVR) in patients receiving AC-T was significantly lower than the baseline. Greater change in post-exercise heart rate recovery (recovery HR) after chemotherapy was observed in those who had received AC-T; the Recovery HR in AC-T patients was significantly higher during post-exercise period than that in CAF patients. Conclusions: AC-T chemotherapy increases RHR and impairs exercise tolerance after chemotherapy more than CAF. Moreover, AC-T also lowers myocardial perfusion more than CAF after chemotherapy.

## 1. Introduction

Breast cancer is the most common and progressive cancer among women globally, and the prevalence of breast cancer has increased by 40% in the last three decades [1,2]. Most patients with early-stage breast cancer receive adjuvant chemotherapy after surgical removal of the lesion, because such intervention has been deemed effective in reducing disease relapse [3,4,5,6,7]. Research conducted by the Early Breast Cancer Trialists’ Collaborative Group confirmed that the rational use of adjuvant chemotherapy is effective in treating survivors with early breast cancer [3]. With effects lasting 15–20 years, adjuvant chemotherapy can reduce breast cancer recurrence and mortality rates by more than 50% [3,7]. Specifically, adjuvant chemotherapy combined with chemotherapeutic agents is generally more effective than single agents alone [3]. 

Anthracyclines and taxanes are the most widely used chemotherapeutic agents to treat patients with breast cancer [4], and they are often used in combination with other agents such as fluorouracil (5-FU) and cyclophosphamide [5,6]. Although beneficial for patients with early-stage breast cancer, adjuvant chemotherapy has considerable side effects (mainly nausea, vomiting, and low white blood cell counts). Furthermore, cardiac damage and other problems may occur with the use of doxorubicin and epirubicin, which are anthracyclines commonly used in chemotherapy for breast cancer [8,9]. For example, a recent longitudinal study by Kirkham and colleagues demonstrated that anthracyclines resulted in tachycardia and hypotension [10]. The side effects of paclitaxel and docetaxel, which are the most widely used taxanes for treating breast cancer, include peripheral nerve damage [11,12]. Although adjuvant chemotherapeutic agents are more effective than single agents alone, the multiple physical and psychological side effects caused by chemotherapy also reduce physical activity [13,14,15] and might result in reduced exercise tolerance [16,17]. 

Several commonly used chemotherapeutic drugs used to treat cancer have been reported to cause cardiovascular problems and even cardiomyopathy [18,19,20]. Chemotherapy with drug combinations (e.g., paclitaxel and anthracyclines) tends to exacerbate cardiovascular system problems [21]. Anthracycline-based chemotherapy can cause a high level of aortic stiffness in patients with breast cancer, which may increase the risk of future cardiovascular diseases [22,23]. The aforementioned side effects have been suggested to affect the cardiopulmonary fitness of patients receiving chemotherapy. Moreover, weight gain is common in patients with breast cancer after chemotherapy treatment; this increases the risk of death in both premenopausal and postmenopausal patients with breast cancer [24,25]. To better control body weight and maintain functional capacity, exercise is an essential and effective modality in this population. However, there is still a lack of investigations focusing on the early changes in exercise tolerance and cardiovascular function after the completion of chemotherapy, which may limit the ability of clinical professionals to prescribe an appropriate exercise program for sustaining quality of life in this population.

Clinically, there are several commonly prescribed chemotherapeutic combinations, including (i) anthracycline plus cyclophosphamide administration followed by taxane injection (AC-T) and (ii) cyclophosphamide mixed with anthracyclines and 5-FU (CAF), after breast cancer surgery. According to the previous literature [8,9,10,18,19,20,21,22,23], these commonly used chemotherapeutic reagents all exhibit variable degrees of impact on future cardiovascular problems. 

While the study of Kirkham et al. clearly demonstrated the cardiotoxic effects of anthracycline, trastuzumab, and left-side radiation on patients with breast cancer [10], it is still unknown whether the cardiovascular responses to exercise challenge in this population, which is receiving different combinations of chemotherapeutic agents are different. Understanding the potentially different cardiovascular responses to exercise challenge is critical because it will provide the foundation for precise exercise prescription for patients receiving different adjuvant chemotherapies. Therefore, the purpose of this study was to investigate and compare the effects of adjuvant chemotherapies with currently used drug combinations (CAF and AC-T) on exercise tolerance and cardiovascular function (e.g., arterial compliance, myocardial perfusion, and central hemodynamic functions) in patients with early-stage breast cancer.

## 2. Materials and Methods

### 2.1. Participants

A total of thirty-four patients with early-stage breast cancer completed this study from 2014 to 2015; 15 patients received administration of CAF chemotherapy and and 19 received AC-T chemotherapy. Patient ages ranged from 33 to 60 years. The chemotherapy options were based on their oncologists’ professional diagnoses and clinical decisions. The inclusion criteria included (1) aged between 30 and 60 years, (2) received diagnoses of stage I or II breast cancer, (3) required adjuvant chemotherapy with CAF or AC-T combinations after surgery (breast-conserving surgery or mastectomy), and (4) capability to follow verbal instructions. The exclusion criteria included (1) mental illness, (2) hypertension (3) diagnosed cardiovascular diseases, (4) smoking, (5) musculoskeletal injuries, (6) receiving chemotherapy before surgery, (7) history of relapsed or metastasized cancers, (8) treated with other cancer therapies at the same time, (8) treated with radiation therapy, or (9) treated with trastuzumab before chemotherapy or added trastuzumab to their chemotherapy drug regimen. The eligible participants provided their written informed consent. All the experimental procedures were approved by the Institution Review Board of Koo Foundation Sun Yat-Sen Cancer Center (Protocol #20140109A) and conducted according to the latest version of the Declaration of Helsinki. 

### 2.2. Study Design

**Figure 1:**
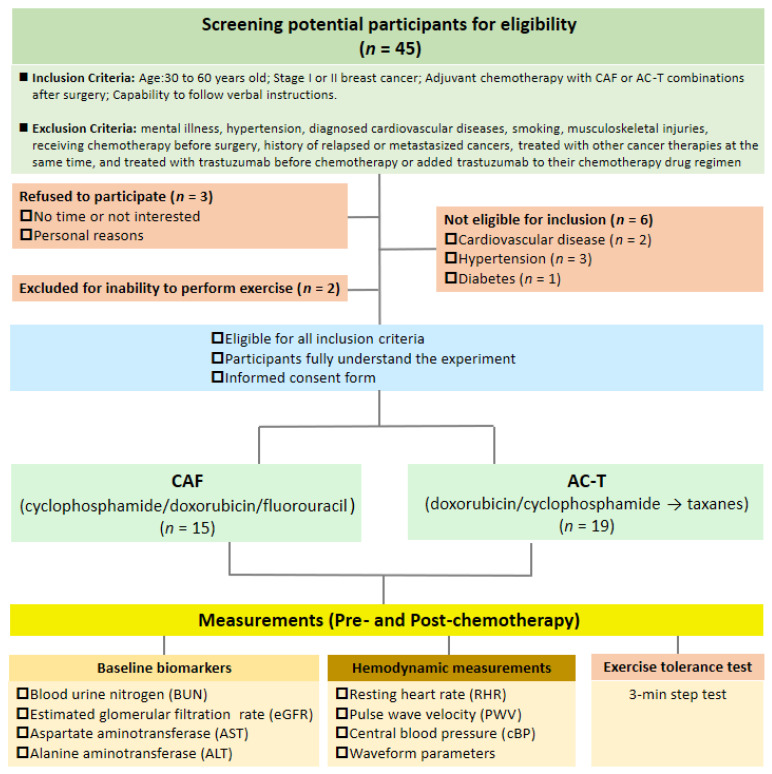
Flow chart of the study participants’ screening and recruitment.

This was an observational study where patients received and completed CAF (*n* = 15) or AC-T (*n* = 19) treatments based on their oncological specialists’ clinical decisions. The baseline outcome measurements (Pre) were performed one week before the first adjuvant chemotherapy session, and the post-intervention measurements (Post) were conducted within two weeks after the last adjuvant chemotherapy session. To ensure consistency, the patients were asked to maintain their lifestyle and avoid participating in additional exercises. They were also asked to abstain from caffeine or alcohol and physical activity above moderate-to-vigorous intensity for at least 48 h before the cardiovascular and exercise tolerance measurements. The flow chart of this study is shown in Figure 1.

### 2.3. Adjuvant Chemotherapy Intervention

The courses for the two different chemotherapies are detailed as follows: Patients in the CAF group received six intravenous injections of cyclophosphamide–anthracycline–5-FU (Doxorubicin). Because injections were administered at 3-week intervals, CAF intervention required at least 15 weeks to complete. Patients in the AC-T group received four cyclophosphamide–anthracycline intravenous injections and four separate taxane injections (doxorubicin + paclitaxel), constituting a total of eight chemotherapy sessions. Because injections were administered at 3-week intervals, AC-T intervention required at least 21 weeks to complete. In this study, CAF and AC-T interventions required an average of 15.1 ± 0.50 and 21.5 ± 0.23 weeks, respectively, to complete. The total accumulative doses of doxorubicin (anthracycline) received by patients in the CAF and AC-T groups were 296.0 ± 4.0 mg/m^2^ (CAF single injection dose: 49.3 ± 0.7 mg/m^2^) and 240.0 ± 0.0 mg/m^2^ (AC-T single injection dose: 60.0 ± 0.0 mg/m^2^), respectively. The total accumulative doses of paclitaxel (taxane) was 255.8 ± 6.2 mg/m^2^ in the AC-T group (single injection dose: 63.9 ± 1.6 mg/m^2^).

### 2.4. Function Measurements

#### 2.4.1. Blood Pressure and Pulse-Wave Velocity (PWV)

An automatic heart-rate and blood-pressure monitor was used to measure the heart rate and blood pressure of participants before and after chemotherapy intervention. Participants were asked to rest in a supine position for 15 min before PWV measurements. Dual-channel photoplethysmography was used to measure PWV [26] (PWV is commonly used for clinically evaluating the elastic properties of the arterial system). Infrared optical sensors were attached to a participant’s right index finger and right toe to measure the pulse wave propagation time (ΔT) between the finger and toe, and a tape measure was used to measure the distance between the right index finger and the right toe (ΔD) of the participant to obtain ΔD/ΔT. This method has adequate reliability and validity in measuring PWV [27]. 

#### 2.4.2. Waveform Analysis

A vascular tonometer (SPT301, Millar Inc., Houston, TX, USA) was used to obtain the radial artery pulse wave. The original pulse wave signal and electrocardiogram were integrated and sent to a physiological signal monitoring system (MP36, Biopac Systems, Inc., Goleta, CA, USA) for later analyses. A customized Matlab program was used to convert the radial artery waveform into a carotid artery waveform to obtain central systolic blood pressure (cSBP), diastolic pressure (cDBP), pulse pressure (cPP), augmented pressure, and augmentation index (AI%). By using the triangulation method, the carotid artery waveform was separated into the incident and reflected waves [28,29], to determine the forward (Pf) and backward (Pb) pressure waves, aortic characteristic impedance (Zc), systolic time (ST; an index of cardiac sympathetic activity) [30], and the subendocardial viability ratio (SEVR) [28]. SEVR, also known as the Buckberg index, has been used as a surrogate measure of myocardial perfusion that correlates with the ratio of subendocardial to subepicardial blood flow, which is indicative of poorer perfusion if the SEVR value is lower than 1.0 [31].

### 2.5. Step-Exercise Tolerance Assessment

The eligible participants performed a modified three-minute bench step test to assess exercise tolerance by measuring the heart rate changes during post-exercise recovery, and the stair-climbing step test was conducted by having the patients step-up/down on a 35-cm height bench at a 96 clicks/minute up-and-down cadence for 3 continuous minutes [32]. Before the step test, the patients were asked to sit and rest for 10 min before measuring their baseline heart rate and blood pressure using a fingertip pulse oximeter (SB220, Rossmax Inc., Taipei City, Taiwan) and a Microlife BP A3 PC (Microlife AG, Widnau, Switzerland), respectively. A physical therapist with cancer treatment specialization allowed the patients to have a short 15-s stepping practice for familiarization, and the physical therapist also closely monitored all participants' work rate and their health conditions during the bench step test. Thereafter, the patients were asked to sit for a standardized post-exercise recovery immediately after the end of step exercise. For assessment of the heart rate response and double-product during the exercise tolerance test, the heart rate and blood pressure were periodically measured at resting baseline, end of step test, 1.5-min post-exercise recovery, and 3 min post-exercise recovery.

### 2.6. Statistical Analyses 

Statistical analysis was performed using SPSS software (v. 23.0, IBM Corporation, Armonk, NY, USA). The distribution normality of the data (Shapiro–Wilk test) was checked before performing further statistical analysis. A two-way repeated measure analysis of variance was used to test the time and treatment differences before and after chemotherapy (Pre- vs. Post-chemo). Moreover, an independent *t*-test was used to compare the changes to chemotherapy between groups. All data are expressed as mean ± standard error of the mean (mean ± S.E.M). *p* < 0.05 was considered statistically significant.

## 3. Results

### 3.1. Characteristics of Participants

The anthropometrical and blood biochemical characteristics of participants with early-stage breast cancer are displayed in Table 1. The average age of a total 34 participants was 44 ± 1 years (range 33–60 years old); CAF: 45 ± 2 years; ACT: 42 ± 2 years). The average participant BMI was 20.8 ± 0.4 kg/m^2^, body fat percentage 28.9 ± 0.8%, and waist circumference 73.9 ± 0.9 cm. There were no significant differences between the CAF group and the AC-T group in all baseline characteristics. Moreover, there were no differences in the blood biochemical markers of hepatic and kidney functions between groups before the chemotherapy.

### 3.2. Central Hemodynamic Changes before and after Chemotherapies

**Figure 2:**
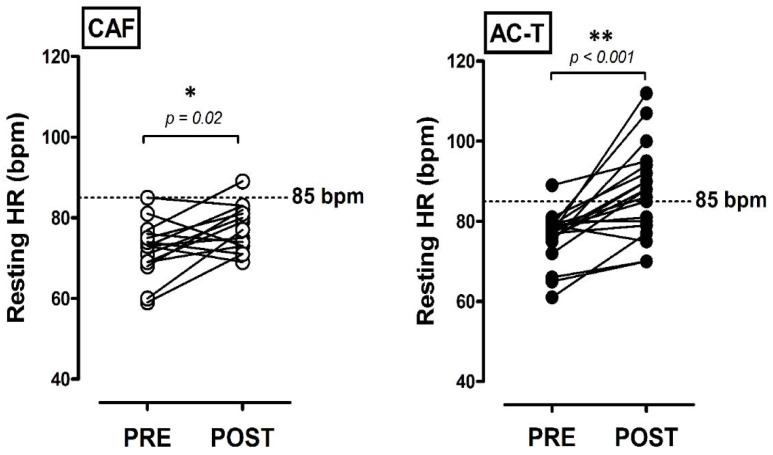
The individual changes in resting heart rate (RHR) with different types of chemotherapy. PRE, baseline before chemotherapy; POST, baseline after the completion of chemotherapy; CAF, cyclophosphamide-anthracycline-5-FU; AC-T, cyclophosphamide-anthracycline + taxane. * denotes the significant difference between PRE and POST (*p* < 0.05). ** denotes the significant difference between PRE and POST (*p* < 0.001).

Table 2 presents the central hemodynamics and measures of waveform analysis. There were no significant time and group differences in cSBP, cDBP, cPP, AI%, or PWV before and after chemotherapies. There were also no significant changes in Pb, Pf, or Zc. SEVR post-AC-T-treatment was significantly lower than the pre- (*p* = 0.008); it was also significantly lower than that of CAF treatment (*p* = 0.05). There were no significant changes in systolic time before or after treatment. However, the AC-T group possessed significantly lower diastolic time after treatment compared with the Pre (*p* = 0.001) and CAF group (*p* = 0.04). Figure 2 illustrates the individual changes in RHR in response to different chemotherapies, both CAF and AC-T increased RHR after treatment (CAF: *p* = 0.02; AC-T: *p* < 0.001). There was only one participant in each treatment group with RHR > 85 bpm before chemotherapy. Only one out of 15 participants had RHR > 85 bpm after CAF administration (6.7%), but there were 12 out of 19 with RHR > 85 bpm after AC-T treatment (63.2%).

### 3.3. Heart Rate Responses to a Step-Exercise Tolerance Test

**Figure 3:**
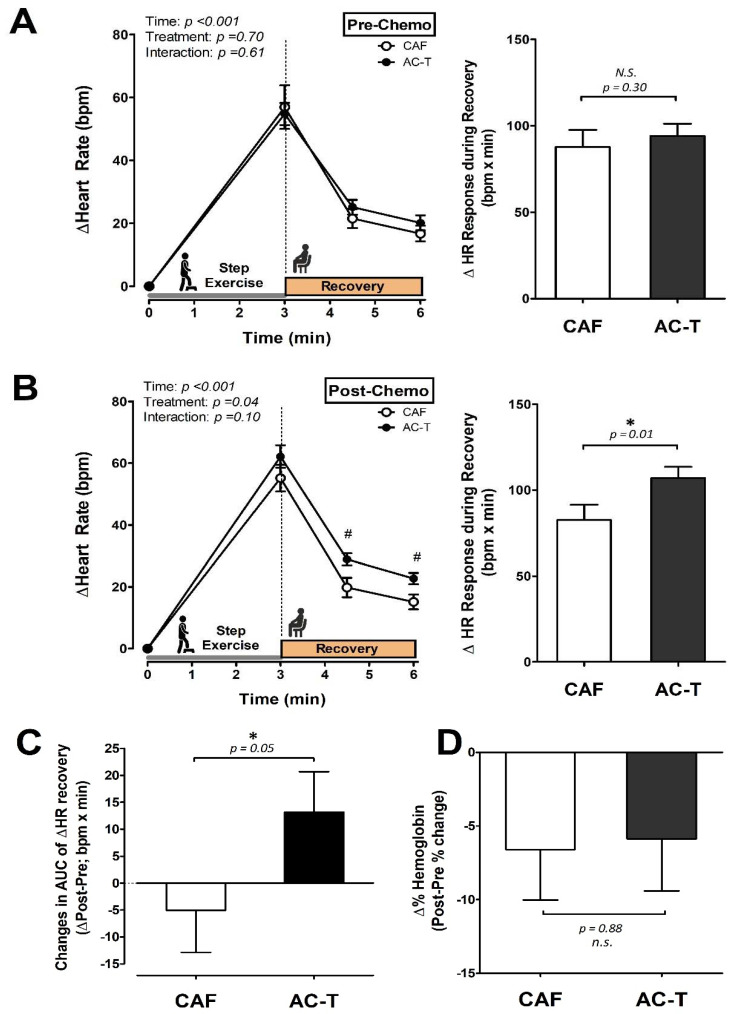
The heart rate responses to a step exercise tolerance test. (**A**) The heart rate responses to the exercise tolerance test and the recovery heart rate AUC (Recovery HR AUC) at the baseline before chemotherapy administration. (**B**) The heart rate responses to the exercise tolerance test and the recovery heart rate AUC (Recovery HR AUC) after the completion of chemotherapy administration. (**C**) The changes in HR recovery AUC between Pre- and Post-chemotherapy. (**D**) The percentage change in the hemoglobin levels between Pre- and Post-chemotherapy. CAF, cyclophosphamide-anthracycline-5-FU; AC-T, cyclophosphamide-anthracycline + taxane. ^#^ denotes the difference between CAF and AC-T using post-hoc test (*p* < 0.05). * denotes the significant difference between Pre- and Post-chemo (*p* < 0.05). Data presented as mean ± S.E.M.

Figure 3A,B illustrate the heart rate (HR) in response to the step exercise and the area under the curve of HR during post-exercise recovery (HR recovery AUC) at Pre- and Post-chemotherapy, respectively. There were no differences in HR changes in response to the step-exercise tolerance test between groups before chemotherapy. However, after the chemotherapy, the AC-T group exhibited greater HR responses than the CAF group during post-exercise recovery at both measured time points (post-1.5 min and post-3 min; *p* < 0.05); moreover, the AC-T group exhibited greater HR recovery AUC than the CAF group after chemotherapy (*p* = 0.01; Figure 3B). In addition, the changes in HR recovery AUC between Pre- and Post-chemotherapy were significantly greater in the AC-T group than in the CAF group (*p* = 0.05; Figure 3C). Moreover, the percentage change in hemoglobin (Hb) levels between Pre- and Post-chemotherapy was not different between AC-T group and CAF groups (*p* = 0.88; Figure 3D).

## 4. Discussion

The primary findings of the present study are as follows. (1) Patients treated with AC-T chemotherapy (accumulative doxorubicin/anthracycline dose: 240.0 ± 0.0 mg/m^2^; accumulative paclitaxel/taxane dose: 255.8 ± 6.2 mg/m^2^) exhibited significantly higher resting heart rate (RHR) compared to patients who received CAF chemotherapy (accumulative doxorubicin/anthracycline dose: 296.0 ± 4.0 mg/m^2^). (2) CAF and AC-T chemotherapies did not result in the development of arterial stiffness when using PWV measurement, but a waveform analysis revealed that the SEVR of patients after AC-T intervention was significantly lower than that before the intervention, indicating that AC-T exerts a greater negative effect on microvascular myocardial perfusion than CAF does. (3) The patients treated with AC-T exhibited significantly greater post-exercise heart rate recovery compared with those who received CAF. 

Here we observed that the RHR was significantly elevated after chemotherapy in both groups, which is in line with the finding of Kirkham et al. (2019) where they found chemotherapy-induced tachycardia peaked eight days after chemotherapy completion in patients with breast cancer [10]. Importantly, the current study demonstrated that the increases in RHR were significantly greater in patients with AC-T than that with CAF (CAF: +6.9% vs. AC-T: +12.6%; Table 1). It has been reported that an RHR > 85 bpm could be defined as asymptomatic tachycardia, which may result in an increased risk of death from future cardiovascular events [33,34,35]. In this study, only one participant had RHR > 85 bpm before chemotherapy. However, approximately 63% (12 out of 19) of the patients with AC-T increased their RHR above 85 bpm after chemotherapy, whereas only about 6.7% (1 out of 15) of patients with CAF exhibited the same pattern (Figure 2). Taken together, chemotherapy increased the RHR of patients, however, the impact was dependent on chemotherapeutic combinations. Chemotherapy-induced asymptomatic sinus tachycardia was more pronounced in patients administrated with AC-T. 

RHR closely reflects the changes in myocardial oxygen consumption, coronary blood flow, autonomic regulation, and myocardial function [36,37]. The greater tachycardia induced by AC-T chemotherapy may have been due to the taxane-induced autonomic nerve impairments, because taxane-induced neuropathy is one of the most limiting side effects of taxane-based adjuvant chemotherapy in patients with breast cancer [12,38,39]. In this regard, we further measured cardiac sympathetic activity using the systolic time (ST) [30] to determine whether autonomic neuropathy was involved, and the results showed that the ST decreased in the AC-T group (approached significance in time effect, *p* = 0.09; Table 2), suggesting a greater increase in cardiac sympathetic beta-adrenergic activity after AC-T treatment. Our findings are in line with finding that chemotherapeutic agents induced autonomic cardiac dysfunctions [39]. In addition, the resting diastole time after AC-T chemotherapy was significantly reduced to a degree lower than that of CAF in the present study (Table 2), suggesting the possible impacts on parasympathetic nerve functions. On the other hand, chemotherapy-induced anemia has been previously suggested to result in fatigue and other functional impairments [40,41,42]; therefore, we then further examined whether AC-T and CAF led to a varied degree of anemia after chemotherapy. Of note, our finding revealed that the post-chemotherapy Hb level in both groups was somewhat lower than the cutoff value (12 g/dL) as suggested [43], but the anemia status was still mild based on the previous report by Kirkham et al. [10]; the reduction of Hb was comparable between chemotherapies in the present study (Figure 3D). This indicates that the potential differences of anemia-induced pathological responses between AC-T and CAF after chemotherapy, such as increased sympathetic activation [44] or impaired exercise capacity, might be excluded, even though both chemotherapy treatments induced a similar degree of minor anemia. Based on our results, however, we still cannot clarify the impact of taxane addition in such impairments. Consequently, although the causes of greater RHR after AC-T treatment than after CAF-based chemotherapy remain unclear, we speculate that chemotherapy-induced autonomic neuropathy or cardiac dysfunction might be involved in both CAF and AC-T chemotherapeutic administrations. However, the AC-T treatment seemed to cause even greater impairments in cardiac regulatory functions in the early stage after chemotherapy.

Additionally, compared with the CAF group, SEVR, a myocardial perfusion index [31], in the AC-T group was significantly reduced after chemotherapy, suggesting that microvascular myocardial perfusion was also compromised, and the extent to which reduced SEVR resulted from chemotherapy was different between treatments (Table 2). Such impairment could be attributed to the reduction of cardiac diastolic function, which could also be explained by the chemotherapy-agent-induced myocardial fibrosis and subsequent cardiac dysfunction in animals [45]. Moreover, SEVR reduction in the AC-T group was unlikely to be associated with body weight and age as both groups tended to increase body weight after the treatment with comparable percent body fat changes; the AC-T group was relatively younger than CAF group. Nevertheless, our findings showed that impaired myocardial perfusion could weaken myocardial oxygen consumption and coronary blood flow, thereby increasing RHR to a greater degree after AC-T treatment.

Although we did not directly determine the degree of cardiomyocyte damage in response to different types of chemotherapy, the existing literature indicates the chemotherapeutic-agent-induced cardiotoxicity and provides explanations for the possible underlying mechanisms of chemotherapy-induced high RHR. For example, among alkylating agents, high-dose cyclophosphamide (approximately 120–200 mg/kg) may severely impair cardiac function through damaging endothelial cells and cardiomyocytes [46,47]. Similarly, taxol-administration-induced asymptomatic ventricular tachycardia has also been reported [48], which further supports our findings of increasing RHR in breast cancer patients. However, both CAF and AC-T contain cyclophosphamide, thus the role of cyclophosphamide in such different cardiovascular impairments between CAF and AC-T can be excluded. Furthermore, the frequently observed combination of paclitaxel and anthracycline has been reported to have even stronger cardiotoxic effects [46,49]. Although the CAF consisted of a higher accumulative dose of doxorubicin/anthracycline than AC-T in this study, we observed that AC-T (with additional paclitaxel) caused greater negative impacts on RHR and other cardiovascular hemodynamic indicators. Therefore, our current results further confirm the previous findings [46,49]. However, considerable variability remains in individual dose-response relationships regarding cardiac toxicity [46,50,51].

Previous studies have reported that patients with breast cancer experience a significant increase in aortic stiffness after receiving anthracycline-based chemotherapy, which may increase the risk of future cardiovascular disease [22,23]. Unlike these previous studies, we found that clinical cardiovascular hemodynamic risk factors such as central and brachial blood pressure, as well as PWV and Pb in both groups, did not change after the chemotherapy, which implies that in the short term, both AC-T and CAF chemotherapy did not appear to exert acute adverse hemodynamic risks (Table 2). Similar to our findings, Souza et al. reported that the hemodynamic parameters associated with arterial stiffness were not changed in the early phase of doxorubicin-based chemotherapy [52]. The discrepancy between our findings and several previous reports may be due to the relatively younger enrolled participants (mean age: 44 years) in this study. Because aging is an independent risk factor for cardiovascular diseases [53], we speculate the negative impacts of chemotherapeutic agents on older patients could be relatively more noticeable. Importantly, our results reveal that the progress between cardiac dysfunction and arterial stiffness in response to chemotherapy might occur in varied time-courses during the early phase of treatments. Nonetheless, the long-term effects of these two adjuvant chemotherapies on arterial stiffness changes in relatively younger patients with breast cancer warrant further research.

The adjuvant chemotherapy of early-stage breast cancer may result in myocardial toxicity causing functional and structural alterations in the cardiovascular system [21,46,47,49,54,55,56], ultimately impairing cardiovascular response during exercise for patients with breast cancer. Additionally, lower endurance capacity has been reported in breast cancer survivors compared with their health- and age-matched counterparts [57]. Therefore, we further assessed exercise tolerance by using a submaximal stepping exercise to observe heart rate changes during and after exercise. We found post-exercise heart rate was significantly higher in the AC-T than that in the CAF group. The post-exercise heart rate recovery, which has been recognized as the indicator of exercise tolerance [58], suggested that exercise tolerance after AC-T treatment was lower than after CAF treatment. Altogether, our findings indicate that the greater magnitudes of reduced diastolic function, reduced myocardial perfusion, and increased heart rate responses during the post-exercise recovery period might be, at least in part, responsible for exercise intolerance following AC-T chemotherapy treatment (Table 2). It appears that these remarkably adverse changes in myocardial dysfunctions may be attributed to cardiotoxicity after chemotherapy, and be associated with exercise tolerance attenuation after AC-T administration in patients with early-stage breast cancer. 

On the other hand, post-exercise heart rate recovery is recognized as a clinically feasible method to quantitatively assess parasympathetic function [59]. Our findings showed that the greater increased post-exercise heart rate recovery after AC-T administration presents the more severe impairment in parasympathetic regulatory function (Figure 3B,C), despite both chemotherapies increasing resting cardiac sympathetic activity (reduced ST; Table 2). Likewise, we also observed that the resting diastolic time was significantly reduced in the patients with AC-T administration, further supporting that the cardiac parasympathetic function was also more substantially perturbed by AC-T (Table 2). These results, therefore, suggest that the AC-T chemotherapy, possibly due to the taxane-induced neuropathy [12,38,39], leads to a greater degree in cardiac autonomic dysfunction exhibited by the greater imbalance of sympathetic/parasympathetic nerve activity [60]. Given this, we herein provide the evidence for and further highlight the importance of the above factors in the greater reduction in exercise tolerance of breast cancer patients receiving AC-T treatment.

Many previous studies have investigated the effects of the safe cumulative dose of taxanes on the development and incidence of long-term heart failure complications [18,19,20,21,61,62]. Therefore, another important finding of this study is that the development rate of taxane-induced cardiotoxicity and the negative impacts on exercise tolerance appeared in the early phase after the end of chemotherapy (Figure 2 and Figure 3). It has to be noted that taxane drugs are still used quite frequently in triple-negative breast cancer patients, regardless of the country or region [63]. Furthermore, we also found that the CAF group actually received an even higher cumulative dose of doxorubicin than the AC-T group; however, the ACT group still showed more severe deterioration in cardiac function parameters and exercise intolerance. These results further confirm that taxane-based chemotherapy produces early-developed negative impacts on cardiac function and exercise tolerance in the patients with early breast cancer, even when they were treated with safe cumulative doses.

***Limitations***: Compared to the other larger clinical trial (49 participants with AC-T treatment) [10], the sample size of this study was relatively small. However, it is to be noted that our study primarily focused on the comparisons of different chemotherapies (AC-T vs. CAF) on the cardiovascular physiological responses to exercise. Thus, the overall recruiting number could be limited because we excluded patients receiving radiation therapy and those with cardiovascular/metabolic diseases to control possible confounding factors. Moreover, the willingness to participate of many potential participants was limited due to their considerations of physical weakness after chemotherapy. Therefore, although we tried our best to recruit participants, we were unable to recruit an even larger number of participants during the recruitment period. Moreover, it has to be noted that the treatment courses of these two chemotherapies were varied, thus the varied treatment periods between AC-T and CAF could be a limitation in the present study. Another limitation of this study is that we did not conduct a randomized and double-blind design, because the selections of chemotherapy were based on oncologists’ clinical decisions. In addition, the provision of chemotherapeutic approaches had to follow the guidance of Taiwan's health insurance system, thus these chemotherapeutic combinations might not be the best choices for the patients. However, our findings still provide an alternative entry-point for clinicians to consider differently their clinical decision when prescribing medications.

***Clinical Implications:*** Although the previous longitudinal study showed that chemotherapy leads to cardiotoxic effects in patients with breast cancer [10], it is still little-known whether chemotherapies using AC-T or CAF differ in cardiovascular impairment and exercise capacity. The obtained results suggest that the patients with breast cancer receiving AC-T required more attention during exercise than their counterparts receiving CAF chemotherapy because AC-T appears to lead to more severe impairments in both cardiac and autonomic nerve dysfunction, which deserves more precautions from clinical care.

## 5. Conclusions

In conclusion, we herein demonstrate that AC-T treatment elicits greater reductions in myocardial perfusion and exercise tolerance compared to CAF chemotherapy in patients with early-stage breast cancer. Furthermore, the impairment of myocardial perfusion appears to occur preceding the systemic arterial stiffening. Our findings, therefore, suggest that the progress between the development stages of cardiac dysfunction and arterial stiffness in response to chemotherapy agents might occur in varied time-courses during the early phase of chemotherapy treatments. Future studies integrating left ventricular ejection fraction (LVEF) or other circulating biochemical markers (e.g., troponin-c) are warranted to better elucidate the underlying mechanisms. Of note, this study provides relevant information regarding the variety of cardiovascular responses to exercise after chemotherapy for clinical personnel in the field of oncological and rehabilitation medicine, which may help clinical professionals to precisely prescribe an appropriate exercise program for sustaining the quality of life in this population.

## Figures and Tables

**Table 1:** Basic anthropometrical and blood biochemical characteristics of participants.

	CAF (*n* = 15)		AC-T (*n* = 19)	
	Before	After	Before	After
Age (y)	45 ± 2	-	42 ± 2	-
Height (cm)	162 ± 6	-	159 ± 7	-
Weight (kg)	56 ± 9	59 ± 10	53 ± 7	57 ± 7
RHR (bpm)	72 ± 7	77 ± 6 *	76 ± 6	87 ± 11 *^#^
BMI (kg/m^2^)	21.3 ± 3.3	22.5 ± 3.3	21.0 ± 2.2	22.6 ± 2.3
Body fat (%)	30.5 ± 5.2	31.4 ± 5.4	28.1 ± 4.1	28.6 ± 3.9
Muscle mass (%)	25.3 ± 2.5	25.1 ± 2.4	26.2 ± 2.0	26.5 ± 2.9
Waist (cm)	75.7 ± 5.9	79.5 ± 5.1	73.3 ± 5.8	77.6 ± 6.3
Hip (cm)	92.0 ± 1.5	95.1 ± 1.4	91.2 ± 1.0	94.8 ± 1.0
Waist-to-hip ratio (WHR)	0.82 ± 0.04	0.83 ± 0.03	0.80 ± 0.05	0.82 ± 0.06
bSBP (mmHg)	115 ± 3	113 ± 4	113 ± 4	112 ± 4
bDBP (mmHg)	89 ± 2	88 ± 3	91 ± 4	88 ± 3
Hemoglobin (g/dL)	12.79 ± 0.43	11.79 ± 0.28 *	12.77 ± 0.39	11.80 ± 0.17 *
BUN (mg/dL)	10.64 ± 0.51	-	9.89 ± 0.63	-
Creatinine (mg/dL)	0.76 ± 0.02	-	0.79 ± 0.03	-
eGFR (mL/min/1.73^2^)	88.27 ± 3.03	-	86.89 ± 3.87	-
AST (U/L)	18.21 ± 1.09	-	19.78 ± 1.60	-
ALT (U/L)	16.27 ± 2.77	-	17.72 ± 2.92	-

Values expressed as mean ± SEM. * *p* < 0.05 vs. Pre; ^#^ *p* < 0.05 vs. CAF. AC-T, doxorubicin + cyclophosphamide→paclitaxel; CAF, cyclophosphamide + doxorubicin + 5-FU; RHR, resting heart rate; BMI, body mass index; WHR, waist–hip ratio; bSPB, brachial systolic blood pressure; bDBP, brachial diastolic blood pressure; BUN, blood urea nitrogen; eGFR, estimated glomerular filtration rate; AST, aspartate aminotransferase; ALT, alanine aminotransferase.

**Table 2:** Characteristics of cardiovascular hemodynamic parameters in participants.

	CAF (*n* = 15)		AC-T (*n* = 19)	
	Before	After	Before	After
cSBP (mmHg)	109 ± 3	107 ± 5	109 ± 5	105 ± 4
cDBP (mmHg)	65 ± 4	72 ± 2	77 ± 4	76 ± 3
cPP (mmHg)	34 ± 3	32 ± 3	32 ± 2	29 ± 2
PWV (m/s)	6.8 ± 0.4	7.4 ± 0.5	7.2 ± 0.5	6.4 ± 0.8
AI (%)	33 ± 5	34 ± 5	29 ± 4	30 ± 2
Augmented pressure (mmHg)	10 ± 1	8 ± 2	9 ± 1	7 ± 1
Forward pressure (mmHg)	21 ± 1	20 ± 2	22 ± 1	20 ± 1
Backward pressure (mmHg)	12 ± 1	11 ± 1	11 ± 1	9 ± 1
Zc (dyne/s/cm^5^)	94 ± 9	92 ± 11	109 ± 7	98 ± 8
SEVR	1.20 ± 0.06	1.11 ± 0.07	1.14 ± 0.06	0.94 ± 0.04 *^#^
Systolic time (ms)	363.9 ± 9.3	370.1 ± 10.9	375.7 ± 18.6	351.1 ± 6.6
Diastolic time (ms)	502.4 ± 21.8	471.4 ± 23.1 *	501.8 ± 39.8	379.7 ± 18.3 *^#^

Values expressed as mean ± SEM. * *p* < 0.05 vs. Pre; ^#^ *p* < 0.05 vs. CAF. cSBP, central systolic blood pressure; cDBP, central diastolic blood pressure; cPP, central pulse pressure; PWV, pulse-wave velocity; SEVR, subendocardial viability; AI, augmentation index; Zc, characteristic impedance.

## Data Availability

The data presented in this study are available upon request from the corresponding authors.
